# Herbo-Mineral Medicine, Cardiogrit Gold, Exhibits Protective Effects in *Caenorhabditis elegans* Model of Doxorubicin-Induced Cardiotoxicity

**DOI:** 10.1155/jt/4609428

**Published:** 2025-07-28

**Authors:** Acharya Balkrishna, Saurabh Bhatti, Meenu Tomer, Sudeep Verma, Rishabh Dev, Anurag Varshney

**Affiliations:** ^1^Drug Discovery and Development Division, Patanjali Research Foundation, NH-58, Haridwar 249405, Uttarakhand, India; ^2^Department of Allied and Applied Sciences, University of Patanjali, Patanjali Yog Peeth, Roorkee-Haridwar Road, Haridwar 249405, Uttarakhand, India; ^3^Patanjali Yog Peeth (UK) Trust, 40 Lambhill Street, Kinning Park, Glasgow G411AU, UK; ^4^Special Centre for Systems Medicine, Jawaharlal Nehru University, New Delhi 110067, India

**Keywords:** *Caenorhabditis elegans*, Cardiogrit Gold, cardiotoxicity, doxorubicin, pharyngeal pumping

## Abstract

Doxorubicin, an effective antineoplastic agent, is often prescribed for the treatment of various carcinomas. However, the use of doxorubicin becomes limited due to its adverse effects like cardiotoxicity, dysmenorrhea, and leucopenia. Cardiogrit Gold (CG) is a herbo-mineral Ayurvedic medicine prescribed for the treatment of various cardiovascular ailments. The current study aimed to investigate the therapeutic potential of CG in imparting protection against doxorubicin-induced cardiotoxicity. Wild-type (N2) and genetically modified *Caenorhabditis elegans*(SJ4005 and DA597) were used as model organisms to assess the bioactivity of CG against doxorubicin-induced cardiotoxicity. Chemical characterization of CG was performed by HPLC-based analysis. Calcium, a key mineral component of CG, was measured in CG-treated *C. elegans* using inductively coupled plasma mass spectrometry (ICP-MS) analysis, as the marker of CG internalization in *C. elegans*. Toxicity induced by doxorubicin and its recovery upon CG treatment was determined by various toxicologically important endpoints. CG treatment rescued N2 *C. elegans* from doxorubicin-induced reduction in their growth, reproduction, locomotory behavior, pharyngeal pumping, feeding ability, and increased ROS generation. CG treatment modulated the expression of hsp-4 in SJ4005 *C. elegans* suggestive of decreased ER stress and normalized the pharyngeal grinder damage in DA597 *C. elegans,* indicating a robust induction of cardio-normalcy. Novel analytical methods were developed to detect and quantify doxorubicin in *C. elegans* on HPLC and UPLC/QToF-MS platforms. Interestingly, CG treatment decreased bioaccumulation of doxorubicin in *C. elegans,* robustly correlating with the observed cardioprotective effects. Taken together, CG has a strong cardioprotective profile against doxorubicin-induced damages and could be taken for further preclinical and clinical assessments.

## 1. Introduction

Cancer is one of the major public health crises worldwide and was responsible for 10 million deaths in 2020 [1]. The use of effective anticancer therapies greatly increases the survival rate of cancer patients. However, cancer-related deaths still occur even after the patients receive appropriate anticancer therapy, largely due to chemotherapy-associated toxicities. Cardiotoxicity is one of the major chemotherapy-associated adverse effects that has been correlated with death, disability, and a decrease in quality of life in cancer patients [2, 3]. In some cases, cardiotoxicity latently manifests long after completion of the chemotherapy [4]. Patients diagnosed with cardiotoxicity are treated with conventional heart failure medications. However, a lacuna still persists for the effective management of cardiotoxicity-related pathologies [5, 6].

Doxorubicin is an antineoplastic agent derived from the *Streptomyces peucetius* bacteria and belongs to the anthracycline group of chemotherapeutic agents. It is widely used for the treatment of tumors and hematological malignancies. However, its adverse effects cause physiological damage to the heart mainly via inducing cardiac dilation, and systolic dysfunction, sometimes leading to cardiac failure [4, 7, 8]. These clinical manifestations are managed by the combined therapeutic use of corticosteroids and statins [9]. However, extensive use of corticosteroids and statins often leads to other comorbidities, like muscle injury and insomnia [10–13]. Hence, an alternative, safe, and widely accessible treatment option is required to cope with the cardio-pathophysiological manifestations of anticancer drugs, like doxorubicin.

The potential of natural products to reduce the harmful effects of chemotherapy on healthy cells without compromising the antitumor activity has been progressively studied [14, 15]. Cardiogrit Gold (CG) is a herbo-mineral prescription medicine for the treatment of heart-related ailments. It is majorly composed of extracts from *Terminalia arjuna* along with classical mineral medicines, *Yogendra Ras*, *Akik Pishti*, *Sangeyasav Pishti*, *Jaharmohra Pishti*, and *Moti Pishti* (Table 1). *Terminalia arjuna* has been widely used as an ethnopharmacological agent for the treatment of clinical conditions like atherosclerosis, myocardial ischemia, cardiomyopathy, and cardiac failure [16]. *Yogendra Ras* is a well-known herbo-mineral medicine used to manage etiologies of cardiac disorders like inflammation and oxidative stress [17]. *Moti pishti,* derived from natural pearls, is a rich source of calcium that plays an important role in the contraction of heart muscles [18].

Herbo-mineral constituents of CG have been described for their therapeutic utilities in cardiac diseases, in the Ayurvedic reference texts, namely, B.P.N.: Bhavaprakasha Nighantu, Edition 2006 and 2010 (Sr. No. 1), B.R: Bhaishajya Ratnavali, 18th Edition (Sr. No. 2), A.S.S.: Ayurved Sar Sangrah, Edition-2010 (Sr. No. 2 to 5), and A.F.I.-II: The Ayurvedic Formulary of India-I, 2nd Edition (Sr. No. 6), on the respective pages, as depicted in Table 1. In addition, *Acacia arabica* (8 mg), microcrystalline cellulose (16 mg), hydrated magnesium silicate (8 mg), and sodium carboxymethyl cellulose (8 mg) have been used for formulating the CG tablet.

*Caenorhabditis elegans* has been used as a model organism to investigate therapies against doxorubicin-induced cardiotoxicity [19]. Although these nematodes do not have a traditional heart-like organ, they do have pharyngeal muscles that have orthologous resemblance with the vertebrate heart. The pharynx of *C. elegans* and the vertebrate heart are both tube-like structures, composed of binucleated muscle cells, with continuous autonomous and rhythmic pumping [20, 21]. The rhythmic contraction and relaxation of the nematode's pharyngeal muscle is termed as *pharyngeal pumping* which is responsible for ingestion and transport of food from mouth to intestine. The *C. elegans* pharynx grinder is required for proper grinding of food (bacteria) and other components before it is passed to the intestine. *Phm-2* mutant worms have damaged or compromised pharyngeal grinder and can be utilized in understanding the uptake of compounds [22].

The present study investigated the potential of CG against doxorubicin-induced cardiotoxicity. Phytometabolite profiling of CG was carried out by HPLC. The safety assessment of CG was done by performing survival and progeny assays in wild-type (N2) *C. elegans*. Doxorubicin-mediated cardiotoxicity in *C. elegans* in the presence and absence of CG was evaluated by analysis of parameters like survival, reproduction, doxorubicin accumulation, growth, pharyngeal pumping behavior, locomotory behavior, feeding ability, and ROS generation in wild-type (N2) *C. elegans*. Additionally, the hsp-4::GFP reporter *C. elegans* SJ4005 strain was utilized for the assessment of ER stress [23], and a *phm-2*(ad597)*C. elegans*DA597 mutant strain was used to identify pharyngeal damage. Overall, this study was conducted to test the pharmacological effects of CG against doxorubicin-induced cardiotoxicity-related etiologies.

## 2. Material and Methods

### 2.1. Reagents

CG (Internal batch# PRF/CHIN/0423/0523) was sourced from Divya Pharmacy, India. HPLC grade Acetonitrile was procured from Finar, India. Methanol, orthophosphoric acid AR grade, dimethyl sulfoxide (DMSO) AR grade, and potassium dihydrogen phosphate AR grade were obtained from Rankem, India. Arjunic acid and arjungenin were purchased from Natural Remedies, India. Doxorubicin was procured from TCI, India. Ellagic acid, 2′,7′-dichlorofluorescin diacetate (H_2_DCFDA), and Tween-20 were obtained from Sigma-Aldrich, USA. Sodium azide was procured from Fisher Scientific, India.

### 2.2. Phytometabolite Analysis of CG

CG (500 mg) powder was diluted to 10 mL in methanol:water (90:10). The mixture was then sonicated for 30 min, centrifuged for 5 min at 10,000 rpm, and filtered by 0.45-μm nylon filter. This filtered solution was used for phytometabolite analysis. Standard stock solutions (1 mg/mL) of ellagic acid, arjunic acid, and arjungenin were prepared in methanol, individually. A standard mix working solution of 50 μg/mL concentration was made by mixing 0.05 mL of each standard and diluting it to 1 mL. The quantification of marker compounds was performed on Prominence-i (LC-2030c 3D Plus, Shimadzu, Japan) HPLC system. The elution was carried out at a flow rate of 1.0 mL/min using a gradient elution of mobile phase A (0.1% orthophosphoric acid) in water (pH 2.5 adjusted by diethylamine) and mobile phase B (acetonitrile). Zorbax Eclipse XDB-C18 (4.6 × 250 mm, 5 μm; Agilent, USA) column was utilized for separation. Gradient programming of the solvent system for mobile phase B was set as 15% for 0 to 5 min, 15% to 20% from 5 to 10 min, 20% from 10 to 15 min, 20% to 40% from 15 to 20 min, 40% from 20 to 25 min, 40% to 80% from 25 to 35 min, 80% from 35 to 40 min, 80% to 15% from 40 to 41 min, and 15% from 41 to 45 min. 10 μL of phytometabolite standard mixture and test solution was injected for the analysis, and column temperature was maintained at 35°C. Wavelength was set at 210 nm for arjungenin and arjunic acid and 365 nm for ellagic acid.

### 2.3. Maintenance of *C. elegans* Culture

The *C. elegans* strains, wild-type N2, SJ4005 [zcls4 (hsp-4::GFP)], DA597 [phm-2(ad597)], and *E. coli* OP50 were purchased from the Caenorhabditis Genetic Center, University of Minnesota, USA. These worms were cultured in nematode growth medium (NGM) plates seeded with *E. coli* OP50, at 20°C [24]. To get synchronized L1 larvae, bleach synchronization was done, which involved treating adult worms with a solution of sodium hydroxide and sodium hypochlorite. The bleach solution dissolves the worm's body, leaving the intact eggs behind. These eggs are resistant to bleach because of their hard shell. The eggs were washed with M9 buffer to neutralize the effect of bleach solution and were allowed to hatch in liquid M9 buffer [25]. These hatched L1 worms were synchronized and used for the study.

### 2.4. *C. elegans* Toxicological Parameters

#### 2.4.1. Survival and Reproduction Assay

CG was initially dissolved in 30% DMSO in PBS, and the final concentration of DMSO was 0.3% for the highest CG concentration (300 μg/mL) tested. Synchronized L1 worms were exposed to CG (1–300 μg/mL) or doxorubicin (0.1–10 μM) in M9 buffer with food for 72 ± 1 h, in the dark along with untreated worms, serving as the control group. After exposure, worms were visually scored using a ZEISS Stemi 305 stereomicroscope (Carl Zeiss, Germany). Percent survival was determined for each treatment concentration, and data were presented as mean ± SEM.

For the reproduction assay, worms were treated with different concentrations of CG (1–300 μg/mL) or doxorubicin (0.1–10 μM) in M9 buffer with food for 72 ± 1 h. After exposure, young adult worms were randomly transferred to new 35-mm NGM plates containing OP50 bacteria (one worm per plate). After 24 h of incubation at 20°C, the parent worm was removed and eggs were allowed to hatch for the next 24 h. After that, L1 progeny were scored in each plate using a ZEISS Stemi 305 stereomicroscope (Carl Zeiss, Germany). Total progeny produced in 24 h per parent worm was determined.

#### 2.4.2. Assessment of Doxorubicin Bioaccumulation in N2 Worms by HPLC

Synchronized L1 worms were exposed to different concentrations of doxorubicin (0.1–10 μM). After 72 ± 1 h treatment, worms were washed with M9 buffer and resuspended in 200 μL MQ water. Subsequently, worms were sonicated for 1 min by probe sonicator, followed by centrifugation at 14,000 rpm for 15 min, and the supernatant was subjected to HPLC analysis and protein estimation using a BCA protein assay kit (G-Biosciences, USA). HPLC analysis was performed by Prominence-XR UHPLC system (Shimadzu, Japan) equipped with a Quaternary pump (Nexera XR LC-20AD XR), DAD detector (SPD-M20 A), Auto-sampler (Nexera XR SIL-20 AC XR), Degassing unit (DGU-20A 5R), and Column oven (CTO-10 AS VP). Separation was achieved using a Shodex-C18 (5 μm, 4.6 × 250 mm) column subjected to gradient elution with a flow rate of 0.8 mL/min. 0.1% trifluoroacetic acid in water (Solution A) and acetonitrile:methanol (8:2) (Solution B). 50 μL of standard and test solution was injected, and wavelength was set at 254 nm. Results were normalized to protein content.

#### 2.4.3. Doxorubicin Exposure and CG Treatment

CG pretreatment was given to synchronized L1 worms at 20°C for 24 h in M9 buffer. Worms were washed and co-treated with different concentrations of CG (1–30 μg/mL) and doxorubicin (10 μM) in the presence of food in M9 buffer until worms reached their adult stage. After co-treatment, different toxicity parameters were studied by analysis of growth, behavior, and reproduction.

#### 2.4.4. Doxorubicin Accumulation and Calcium Deposition

After co-treatment, worms were washed with M9 buffer and resuspended in 200 μL MQ water. Subsequently, worms were sonicated for 1 min by probe sonicator, followed by centrifugation at 14,000 rpm for 15 min. The supernatant was used for UPLC/QToF-MS analysis and protein estimation. Analysis was performed on Xevo G2-XS QToF coupled with Acquity UPLC-I Class (Waters Corporation, USA). The isocratic elution of mobile phase A (0.1% formic acid in water) and mobile phase B (0.1% formic acid in acetonitrile) at a flow rate of 0.3 mL/min with Waters Acquity UPLC HSS T3 (2.1 × 100 mm, 1.8 μm) column was used for analysis. The column temperature was maintained at 27°C, and the sample temperature was maintained at 15°C during analysis. The isocratic elution was set as follows: 25% B for 0 to 8 min. 5 μL of standard and test solution was injected.

Further uptake and accumulation of CG (containing calcium-rich components) was quantified by determining the calcium concentration inside worms. After co-treatment, worms were washed with M9 buffer and resuspended in 250 μL MQ water. Later, worms were sonicated for 1 min by probe sonicator, followed by centrifugation at 14,000 rpm for 15 min. The supernatant was used for calcium analysis, and values were normalized with protein levels. The samples were analyzed for a total calcium concentration using ICP-MS (Model: iCAPRQ, ThermoFisher Scientific, USA).

Furthermore, doxorubicin accumulation was quantified by measuring the red color of doxorubicin within worms using brightfield microscopy. CG co-treated N2 worms were washed and anesthetized with 125 mM sodium azide (NaN_3_) to inhibit the movement of worms. Subsequently, images were captured at 200× magnification using an Olympus BX43 microscope equipped with a Mantra imaging platform (PerkinElmer, USA) and further processed on the Inform 2.2 software suite (PerkinElmer, USA). Quantification of doxorubicin in the head and intestine region of 10 worms was performed from the microscopic images using ImageJ software (NIH, USA) [26].

### 2.5. Growth and Reproduction Assay

The CG and doxorubicin co-treated worms were washed and treated with 125 mM sodium azide (NaN_3_) to inhibit the movement of worms. Furthermore, images were captured at 100× magnification using an Olympus BX43 microscope equipped with a Mantra imaging platform and then processed on the Inform 2.2 software suite. The growth of worms was measured as the mean length of the worm, and analysis was carried out using ImageJ software. The experiment was repeated thrice with 10 replicates for each group [27].

For the reproduction assay, co-treated worms were washed and transferred to new 35-mm NGM plates containing *E. coli* OP50 bacteria (one worm per plate). After every 24 h of incubation at 20°C, worms were transferred into new NGM plates, where they laid eggs. These eggs were incubated further for 24 h to hatch. The total cumulative progeny produced per worm was determined by scoring L1 progeny in each plate using a ZEISS Stemi 305 stereomicroscope (Carl Zeiss, Germany). The experiment was repeated thrice with 3 replicates for each group [28].

### 2.6. Locomotion (Head Thrash) Behavior, Pharyngeal Pumping, and Feeding Ability

Post-treatment, locomotory behavior in N2 worms was monitored using ZEISS Stemi 305 stereomicroscope (Carl Zeiss, Germany) by scoring head thrash frequency [29]. A head thrash is represented by a change in the direction of bending at the midbody. A total of 10 treated worms were harvested and transferred onto a fresh NGM plate. After 1-min relaxation phase, a video of 1 min was shot using ZEISS Stemi 305 stereomicroscope (Carl Zeiss, Germany). Individual worms were manually scored for head thrashes using the recorded video. The experiment was repeated thrice with 10 replicates for each group.

The nematode behavior was monitored under the ZEISS Stemi 305 stereomicroscope (Carl Zeiss, Germany) for pharyngeal pumping. During feeding, worms suck bacteria and grind them in their terminal bulb by muscle contraction. The complete cycle of contraction and relaxation of the terminal bulb is called a pump [30]. The number of pumps/min indicates the feeding behavior of the worm. CG-treated worms were harvested, and 10 worms were transferred onto a seeded NGM plate. After 15-min recovery period, worms were manually scored for pumps/min under a ZEISS Stemi 305 stereomicroscope (Carl Zeiss, Germany). The experiment was repeated thrice with 10 replicates per group.

The feeding ability of worms was quantified by exposing ∼1000 worms in a liquid medium (*E. coli* suspended in M9 buffer with or without co-treatment of CG and doxorubicin) and the change in optical density (OD) of the suspension at 0 and 48 h was measured via absorbance measurement at 600 nm using Envision multimode plate reader (PerkinElmer, USA). The difference in feeding ability was represented as fold change reduction in comparison with that of the control worm. Higher OD of *E. coli* in the suspension represents a lower feeding ability of the worms [31].

### 2.7. hsp-4::GFP Expression and ROS Generation

The hsp-4::GFP expression was assessed in CG and doxorubicin co-treated transgenic SJ4005 worms. After co-treatment, young adult worms were washed and images were captured using FITC filters by Olympus BX43 microscope equipped with a Mantra imaging platform and further processed on Inform 2.2 software suite. Images of 10 individual worms were taken at 200× magnification and quantified using ImageJ software. To determine reactive oxygen species (ROS), ∼1000 worms were treated with different concentrations of CG and worms were then transferred to each well of a 96-well plate. Worms were incubated with 0.05 mM H2DCF-DA (Sigma, USA) for 30 min on an orbital shaker and generation of ROS was measured at 485/535 nm wavelength using an Envision multimode plate reader (PerkinElmer, USA). H2DCFDA is a cell-permeable nonfluorescent dye. Upon entering the cell, it undergoes intracellular oxidation and de-esterification, resulting in the formation of highly fluorescent 2,7-dichlorofluorescein [32]. The fluorescence values were recorded, and the experiment was repeated thrice. The pharyngeal grinder damage was observed using DA597 mutant worms. CG (1–30 μg/mL) treated DA597 worms were washed, and images of their pharynx were analyzed.

### 2.8. Statistical Analysis

Data were expressed as mean ± SEM of three experiments, and the level of significance relative to unexposed control and doxorubicin was determined through one-way or two-way analysis of variance (ANOVA) followed by Dunnett's multiple comparisons post hoc test. GraphPad Prism 8 was used to execute statistical calculations. The value of *p* < 0.05 was considered as statistically significant.

## 3. Results

### 3.1. Phytometabolite Analysis of CG

**Figure 1:**
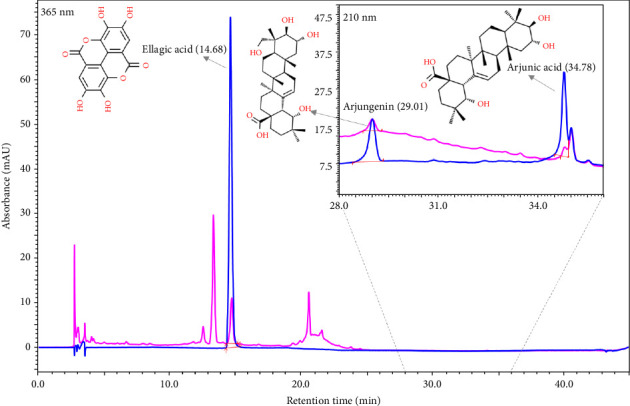
HPLC-based identification and quantification of phytometabolites present in Cardiogrit Gold (CG). Overlayed HPLC chromatograms of standard mix (blue color) and CG (pink color). Ellagic acid was quantified at 365 nm wavelength, and arjungenin and arjunic acid were quantified at 210 nm wavelength. Quantitative analysis of CG is shown in Table 2.

The quantitative analysis of phytometabolites using standard marker compounds confirmed the presence of various analyte peaks at different retention times. Ellagic acid (RT: 14.68 min), arjungenin (RT: 29.01 min), and arjunic acid (RT: 34.78 min) were detected and quantified in CG (Figure 1). Ellagic acid was the most abundant phytometabolite present in CG, followed by arjungenin and arjunic acid (Table 2).

### 3.2. Dose Selection of CG in *C. elegans*

**Figure 2:**
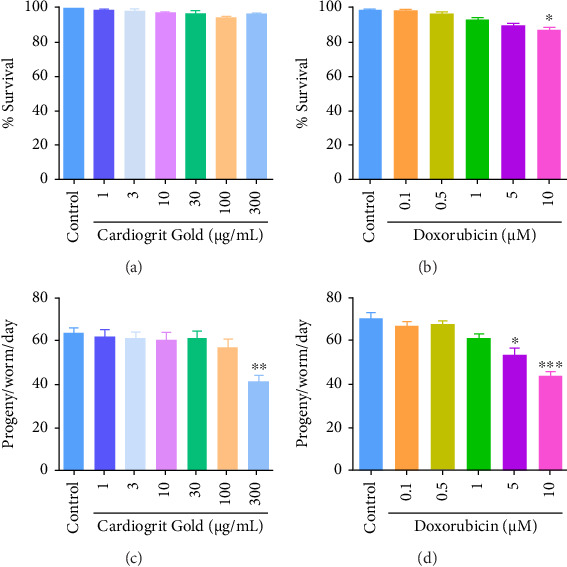
Effect of CG and doxorubicin on survival of N2 worms. (a) CG (1–300 μg/mL), (b) doxorubicin (0.1–10 μM), (c, d) effect of CG or doxorubicin on reproduction. ^∗^*p* < 0.05; ^∗∗^*p* < 0.01; ^∗∗∗^*p* < 0.001 in comparison with control.

For dose selection, N2 L1 worms were treated with different concentrations of CG (1–300 μg/mL) or doxorubicin (0.1–10 μM). CG (1–300 μg/mL) showed no loss in survivability even at 300 μg/mL (Figure 2(a)). However, significant (*p* < 0.05) survival loss was observed at 10 μM doxorubicin concentration, in comparison with untreated control (Figure 2(b)).

**Figure 3:**
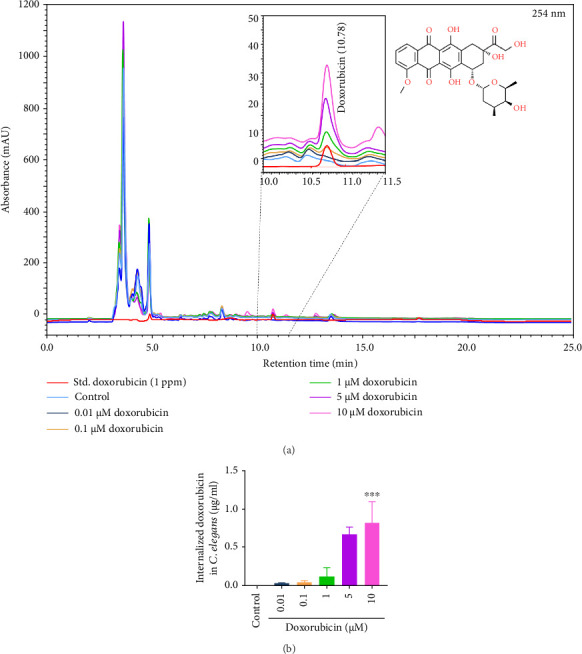
Accumulation of doxorubicin in N2 worms. (a) Overlayed HPLC chromatogram of pure doxorubicin standard, control untreated worms, and varying concentrations of doxorubicin (0.01–10 μM)-treated worms, structure of doxorubicin was sourced from ChemSpider (ChemSpider ID: 29400). (b) Bar diagram of doxorubicin accumulation. ^∗∗∗^*p* < 0.001 in comparison with control.

For further validation, the reproductive toxicity of CG (1–300 μg/mL) or doxorubicin (0.1–10 μM) was evaluated on N2 L1 worms. Except at 300 μg/mL (*p* < 0.01), CG did not affect the progeny production (Figure 2(c)). However, a significant (*p* < 0.01) reduction in the number of progeny per worm was found at 5 and 10 μM doxorubicin concentration compared to untreated control (Figure 2(d)). In parallel, we found a significant (*p* < 0.001) accumulation of doxorubicin at 5 μM (0.66 ± 0.05 μg/mL) and 10 μM (0.82 ± 0.16 μg/mL) concentrations in N2 worms (Figures 3(a) and 3(b)). The concentrations of CG (1–30 μg/mL) or doxorubicin (10 μM) were used for further analysis.

### 3.3. CG Reduces Accumulation of Doxorubicin in N2 Worms

**Figure 4:**
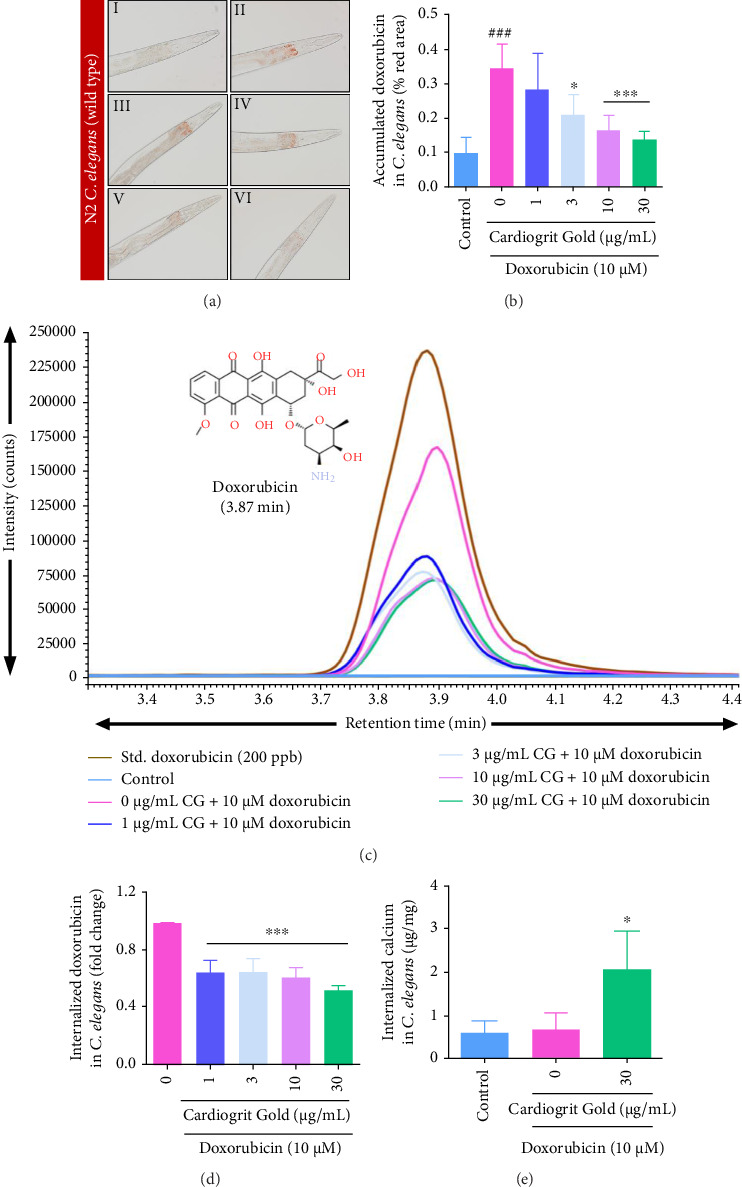
CG decreased the uptake and accumulation of doxorubicin in N2 worms. (a) Brightfield images of *C. elegans* pharynx (magnification = 200×) (I) untreated control, (II) doxorubicin (10 μM), (III–VI) worms co-treated with doxorubicin (10 μM) and varying concentrations of CG (1–30 μg/mL). (b) Doxorubicin red intensity was quantified by ImageJ software. (c) Overlayed UPLC/QToF-MS chromatogram of the reference standard (200 ppb), control worms, 10 μM doxorubicin alone, and co-treatment with CG (1–30 μg/mL). (d) CG (1–30 μg/mL) co-treatment reduced the accumulation of doxorubicin in *C. elegans* as detected by UPLC/QToF-MS. (e) CG (30 μg/mL) increased calcium accumulation in N2 worms as detected by ICP-MS. ^∗^*p* < 0.05 and ^∗∗∗^*p* < 0.001 in comparison with doxorubicin; and ^###^*p* < 0.001 in comparison with control.

In *C*. *elegans* exposed to doxorubicin (10 μM), bright red color accumulation was observed in the pharynx and intestine region which indicates deposition of doxorubicin [33–35]. Co-treatment with CG (3, 10, and 30 μg/mL) significantly (*p* < 0.001) lowered doxorubicin deposition in the pharynx and intestine of the worms in a dose-dependent manner (Figures 4(a) and 4(b)). This observation was further validated by LCMS-based analysis of doxorubicin levels. Likewise, it was also observed that co-treatment of N2 worms with CG (1–30 μg/mL) significantly (*p* < 0.001) reduced doxorubicin accumulation (Figures 4(c) and 4(d)). Parallelly, another correlation was observed between the intracellular levels of calcium and CG treatment, by ICP-MS-based analysis. Doxorubicin-treated worms in the presence of CG (30 μg/mL) displayed a significant (2.08 ± 0.51 fold; *p* < 0.05) increase in calcium concentration (Figure 4(e)). Taken together, CG treatment might regulate the bioaccumulation of doxorubicin.

### 3.4. CG Prevents Doxorubicin-Mediated Reduction in the Growth and Progeny in *C*. *elegans*

**Figure 5:**
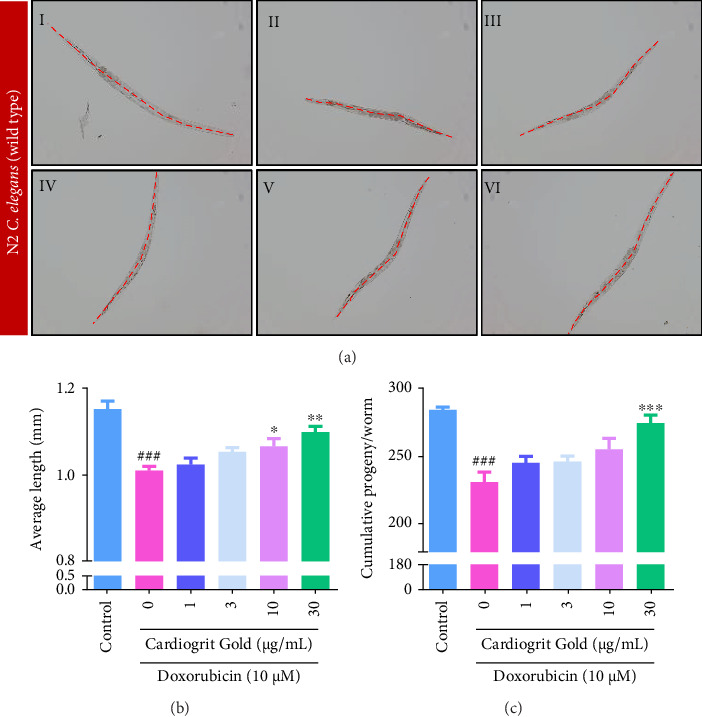
CG enhanced the growth of N2 worms. (a) Brightfield images of *C. elegans* (magnification = 100×) (I) control, (II) doxorubicin, (III–VI) worms co-treated with doxorubicin (10 μM) and varying concentrations of CG (1–30 μg/mL). (b) CG (1–30 μg/mL) increased the length of N2 worms. (c) CG recovered the reproductive ability of the worms. ^∗^*p* < 0.05; ^∗∗^*p* < 0.01; and ^∗∗∗^*p* < 0.001 in comparison with doxorubicin and ^###^*p* < 0.001 in comparison with control.

The mean length of the control worm was found to be 1.15 ± 0.08 mm, whereas, in the worms exposed to doxorubicin (10 μM), the growth was significantly (0.97 ± 0.01 mm; *p* < 0.01) reduced by nearly 16%. CG co-treatment at 10 and 30 μg/mL significantly (*p* < 0.05) recovered the length of N2 worms to 1.07 ± 0.02 and 1.10 ± 0.02 mm, respectively (Figures 5(a) and 5(b)). Furthermore, upon evaluating the effect of doxorubicin on reproduction by counting progeny per worm, it was observed that compared to control (284.88 ± 1.97), doxorubicin exposure significantly (*p* < 0.01) reduced the average progeny by 18.92% (231 ± 7.57). This decline in progeny significantly (275 ± 5.93; *p* < 0.01) recovered in *C*. *elegans* co-treated with CG (30 μg/mL) (Figure 5(c)).

### 3.5. CG Recovers Behavior and Feeding Ability in *C*. *elegans*

**Figure 6:**
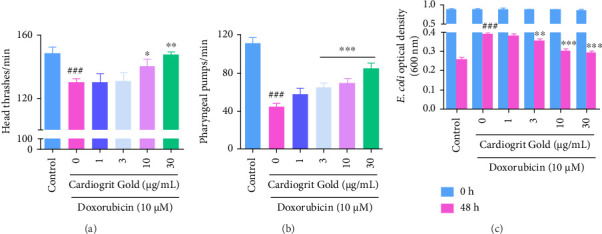
Effect of CG on the behavior of N2 worms. CG (1–30 μg/mL) recovers (a) locomotory behavior, (b) pharyngeal pumping behavior, and (c) feeding ability in N2 worms. ^∗^*p* < 0.05; ^∗∗^*p* < 0.01; and ^∗∗∗^*p* < 0.001 in comparison with doxorubicin and ^###^*p* < 0.001 in comparison with control.

Doxorubicin exposure altered the behavioral parameters like head thrashing, pharyngeal pumping, and feeding ability in N2 worms. Compared to control worms (148.73 ± 3.47), doxorubicin (10 μM)-exposed worms displayed a significant (130.07 ± 2.33; *p* < 0.001) reduction in head thrash behavior (head thrashes per min) by 12.55%. CG co-treated worms displayed significant (*p* < 0.001) recovery in head thrash behavior at 10 μg/mL (140.43 ± 4.23) and 30 μg/mL (147.36 ± 1.81) (Figure 6(a)).

For analysis of pharyngeal pumping behavior, assessment of contraction and relaxation of the terminal bulb is performed by measurement of pump/min displayed by the worm [36]. In doxorubicin-exposed worms, a significant (44.7 ± 1.19; *p* < 0.001) reduction in pharyngeal pumping behavior was observed compared to control worms (111.6 ± 1.98). However, CG co-treated worms at 3, 10, and 30 μg/mL dose showed a significant (*p* < 0.001) recovery in pharyngeal pumping behavior with 65.3 ± 1.52, 70 ± 1.42, and 85.5 ± 1.71 pump/min, respectively (Figure 6(b)).

The feeding ability of *C. elegans* was determined based on the change in OD of the food (*E. coli*) per ∼1000 worms at 48 h, compared to the baseline OD of *E. coli* at 0 h for all the treatment groups. After 48 h co-treatment with CG (3, 10, and 30 μg/mL), the feeding ability significantly (*p* < 0.001) enhanced, as observed from the reduction in OD of *E. coli* left in the plate (Figure 6(c)). Collectively, CG enhanced mobility and feeding ability in worms induced with doxorubicin.

### 3.6. CG Recovers Oxidative Stress and Reduces hsp-4 Expression in SJ4005 *C*. *elegans*

**Figure 7:**
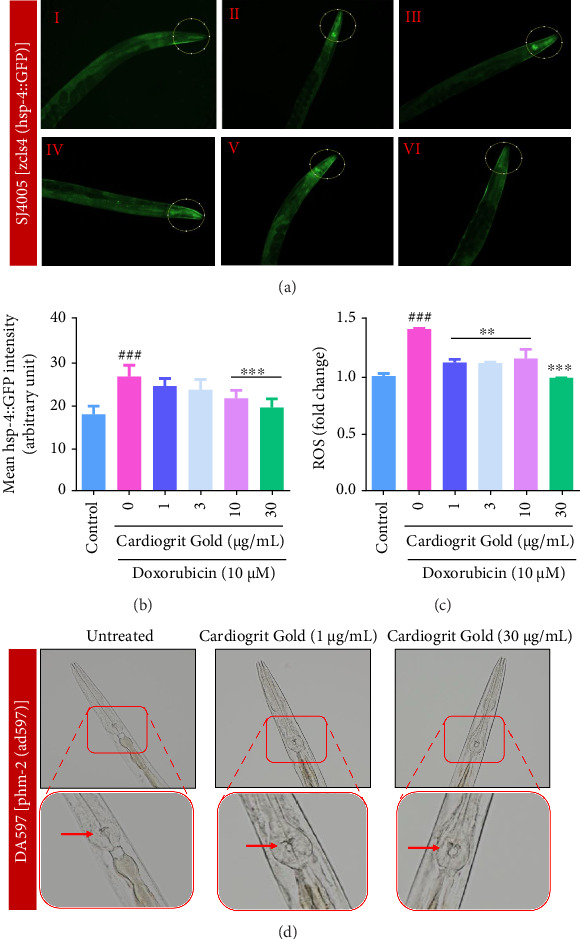
CG recovers GFP intensity in SJ4005 worms. (a) CG (1–30 μg/mL) reduced hsp-4::GFP expression localized in the pharynx of SJ4005 worms (magnification = 200×), (I) control, (II) doxorubicin, (III–VI) worms co-treated with doxorubicin (10 μM) and varying concentrations of CG (1–30 μg/mL). (b) Bar diagram of mean fluorescence intensity of hsp-4::GFP as quantified by ImageJ software. (c) CG treatment decreased the ROS generation (fold change) in doxorubicin-exposed worms. (d) CG (1 and 30 μg/mL) recovers pharyngeal grinder damage in DA579 worms (magnification = 200×). ^∗∗^*p* < 0.01 and ^∗∗∗^*p* < 0.001 in comparison with doxorubicin and ^###^*p* < 0.001 in comparison with control.

In normal conditions, SJ4005 worms express low levels of hsp-4::GFP (marker for ER stress). But its expression increases in response to oxidative stress-induced ER stress [37]. In doxorubicin (10 μM)-exposed SJ4005 worms, bright hsp-4::GFP fluorescence was observed at anterior pharynx region. Interestingly, CG (10 and 30 μg/mL) co-treatment significantly (*p* < 0.001) suppressed the doxorubicin-induced expression of hsp-4::GFP in the pharynx of worms (Figures 7(a) and 7(b)). Parallelly, oxidative stress in N2 worms was also quantified by measurement of the fluorescence intensity of DCFD which enhances with increase in ROS generation. Doxorubicin (10 μM) exposure showed a significant (1.414 ± 0.006 fold; *p* < 0.001) increase in ROS generation compared to control worms. Furthermore, CG (1, 3, 10, and 30 μg/mL) co-treatment significantly (1.118 ± 0.028, 1.115 ± 0.008, 1.155 ± 0.08, 0.9851 ± 0.009 fold; *p* < 0.01) reduced the ROS levels (Figure 7(c)).

DA597 (phm-2 mutant) *C*. *elegans* have a defective pharynx in which the pharyngeal grinder is damaged [20, 38]. Recovery in the damaged pharyngeal grinder of DA597 mutant worms was observed upon CG (30 μg/mL) treatment (Figure 7(d)). Thus, CG is effective against oxidative stress induced by doxorubicin and also recovers damaged pharyngeal grinder which is an orthologous organ to the vertebrate heart [20, 21].

## 4. Discussion

Doxorubicin is a potent chemotherapeutic drug, widely employed in the treatment of various types of cancer, such as leukemia, lymphoma, and other carcinomas. However, despite its effectiveness, the use of doxorubicin is associated with a range of adverse effects [7]. The current study aimed to analyze the cardioprotective efficacy of ayurvedic prescription medicine CG. The quantitative analysis of phytometabolites present in CG by UHPLC revealed the presence of ellagic acid, arjungenin, and arjunic acid. Arjunic acid, a key active ingredient of *Terminalia arjuna*, is well-known for its therapeutic benefits as a cardioprotective agent [39]. Furthermore, arjungenin and ellagic acid have strong free radical scavenging abilities and protect against alterations by heart disease [39–41].

Prior to the evaluation of the efficacy of CG as a cardioprotective drug, the optimum concentration of CG was determined based on survival and reproduction data for CG-treated *C. elegans* to remove any confounding bias. CG was found to be safe up to 100 μg/mL and did not alter the survival and reproductive ability of the worms. Previous studies have shown that doxorubicin accumulates in the pharynx and intestine of the *oct-2* mutant and wild-type N2 worms [33–35]. Similarly, in the current study, a dose-dependent accumulation of doxorubicin was observed majorly in the pharynx region of N2 worms. CG treatment decreased the accumulation of doxorubicin in pharynx in a dose-dependent manner. Interestingly, it was observed that this decrease in bioaccumulation of doxorubicin was inversely proportional to the increase in accumulated calcium levels. Calcium plays a crucial role in contraction (pumping) of *C. elegans* pharynx. It is well documented that increase in the calcium concentration directly induces the rhythmic contractions of the *C. elegans* pharyngeal muscles, which resembles the vertebrate cardiac action potentials [42, 43]. Therefore, CG-mediated beneficial effects could possibly be due to the improvement in the calcium homeostasis. This effect of CG could be attributed to the presence of *Moti Pishti* (herbally processed powdered natural pearls). *Terminalia arjuna* extract has also been known to contain calcium salts that may help in maintaining cardiac muscle contraction by calcium-dependent signaling in the heart [16, 18].

Doxorubicin exposure decreased body length and induced reproductive toxicity. Previous studies have observed that doxorubicin exposure causes amenorrhea in females and leads to the higher risk of heart failure in women with infertility [44, 45]. In the present study, doxorubicin-exposed *C. elegans* showed a reduction in body length and progeny production, and CG treatment restored these parameters to normal. This might be due to the presence of bark extract of *Terminalia arjuna*, which has been extensively reported to possess cardioprotective properties [16] and the ability to improve fertility [46].

In *C. elegans,* major striated muscles are located in the body wall and are necessary for locomotion in liquid or on semisolid surfaces [30]. In some in vivo models, it was observed that doxorubicin exposure reduced the locomotory behavior, such as an unsynchronized swimming pattern in Zebrafish, and locomotory activity in an open field test in rats [47, 48]. Here also, doxorubicin exposure decreases the head thrash locomotory behavior of *C. elegans* in liquid medium which was not observed in CG-treated worms. One possible explanation for this could be the inclusion of *Yogendra Ras* (component of CG), which is known to strengthen the entire body and help with periodic paralysis [17, 49].

Another important consideration in the current investigation was the apparent decrease in pharyngeal behavior observed in *C. elegans* after doxorubicin treatment. Pharyngeal pumping in *C. elegans* correlates with their feeding behavior [50]. Pharyngeal muscle damage might slow the contraction and relaxation of terminal bulb, reducing the amount of food transported from the mouth to the gut [51]. This mechanism resembles bradycardia, a condition in which the heart muscle gets thickened after injury, and results in a difficulty in pumping [20]. The number of contractions of the pharynx's posterior bulb in a minute can be used to quantify the pharyngeal pumping behavior in worms. It was observed that CG treatment recovered pharyngeal pumping behavior in doxorubicin-treated N2 worms. These findings were further validated using *phm-2*-mutant worms which have inherent defects in their pharyngeal grinder that results in increased bacterial accumulation in the intestine. The other *C. elegans* mutants with defects in their pharynx including *eat-2* and *oct-1* have also been shown to possess increased accumulation of doxorubicin inside their pharynx [35]. In the present study, it was established on the functional level that CG might reduce the doxorubicin levels probably by improving the grinder function of *phm-2* mutant or by some other mechanism related to pharyngeal pumping. This might be due to the inclusion of *Jaharmohra Pishti, Akik Pishti*, and *Sangeyasav Pishti* as components of CG, which are known to relax and provide strength to cardiac muscles. Oxidative stress plays a major role in doxorubicin-induced cardiotoxicity. Studies have linked excessive free radicals generated by doxorubicin to the induction of oxidative stress in cardiac cells, leading to its cardiotoxicity [52, 53]. CG-mediated decrease in the levels of ROS suggests its potential to overcome the doxorubicin-induced cardiotoxicity by regulating oxidative stress. The component of CG, *Yogendra Ras*, which contains *Swarna Bhasma* (gold nanoparticles), is known to inhibit ROS generation and could be responsible for its antioxidant effects [17, 54], in addition to the well-known antioxidant effects of *Terminalia arjuna* [55].

Cells produce heat-shock proteins when exposed to different damaging conditions including heat shock, oxidative stress, anticancer drugs, and other factors [56]. Doxorubicin-induced cardiac damage can disrupt the balance between the ER protein load and folding capacity, resulting in the buildup of misfolded or unfolded protein within the ER lumen [57, 58]. It was observed that doxorubicin-induced ROS generation and overexpression of ER stress marker *hsp-4*, in SJ4005 worms, expressing GFP fused to the ER stress marker protein hsp-4. These results can be linked to a previous study wherein the expression of ER stress-related proteins was enhanced in doxorubicin-treated H9c2 cells [58]. Another study linked to ER stress has shown a decrease in the activity of antioxidant enzymes in the heart of doxorubicin-treated mice.

DNA damage appears to play an important early role in anthracycline-induced lethal cardiac myocyte injury [59]. Previous reports suggested that *Terminalia arjuna* bark extract which is a main component of CG has been found to ameliorate various impairments associated with DNA damage and free radical formation on rat adrenal PC-12 cells [60]. Additionally, the extract of *T. arjuna* bark has the potential to inhibit sperm DNA damage caused by cigarette smoking [46]. Ellagic acid, a phytochemical present in CG, is very effective in preventing oxidative DNA damage both in vitro and in vivo [61]. In the lines with these reports, it is conceivable that CG treatment may interfere with the DNA damaging adverse effects of doxorubicin.

**Figure 8:**
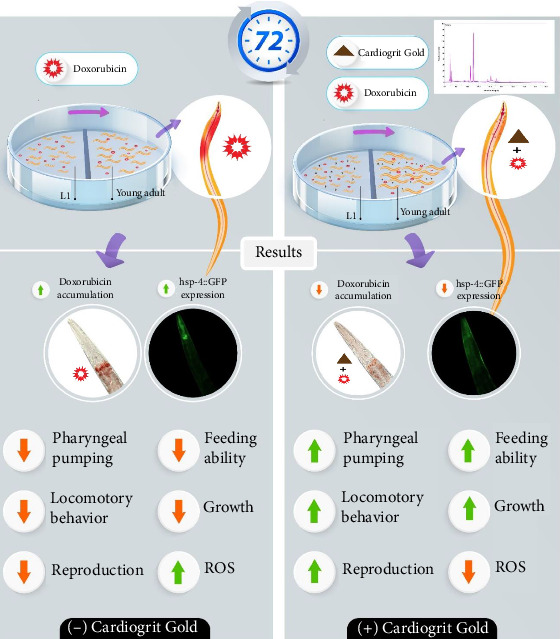
Summary of the protective effect of CG against doxorubicin-induced cardiotoxicity in *C. elegans*.

One potential constraint of the study might be the assumption of pharynx as equivalent to the vertebrate heart. Although the pharynx in *C. elegans* and human heart shares structural and molecular similarities, the pharynx's functions in *C. elegans* might not accurately represent how the vertebrate heart works and needs further evaluation in higher model organisms to enhance the translational value of CG. Collectively, treatment of CG is effective in protecting the worms against doxorubicin-induced ER stress. The outcome of the current study has been summarized in Figure 8.

## 5. Conclusion

CG has shown pharmacological effects to curtail cardiotoxicity, a major adverse effect of chemotherapeutic agents like doxorubicin. The physiological parameters of *C*. *elegans* that mimic the pathophysiological manifestations of cardiotoxicity were observed to normalize upon CG treatment. Parameters, namely, doxorubicin accumulation, growth, behavior, pharyngeal pumping, feeding ability, and oxidative stress which were altered by doxorubicin exposure, were regularized by CG, in a dose-dependent manner. Taken together, CG is a potential candidate for the treatment of chemotherapy-induced cardiotoxicity. This study warrants further clinical investigation of CG in various cardiovascular disorders.

## Figures and Tables

**Table 1:** Composition of CG.

Sr. no.	Common name	Description	Classical book ref.	Page no.	Quantity (mg/CG tablet)
1	Arjuna bark extract	*Terminalia arjuna*	B.P.N	523–524	198

2	*Yogendra Ras*	Classical preparation	B.R.	545	9
			A.S.S.	377	

3	*Akik Pishti*	Classical preparation	A.S.S.	93–94	79

4	*Sangeyasav Pishti*	Classical preparation	A.S.S.	173–174	79

5	*Moti Pishti*	Classical preparation	A.S.S.	145–147	16

6	*Jaharmohra Pishti*	Classical preparation	A.F.I.-II	201	79

**Table 2:** Quantitative analysis of phytometabolites in CG on HPLC platform, as shown in Figure 1.

Sr. no.	Phytometabolite	HPLC retention time (min)	Content in CG (μg/mg)
1	Ellagic acid	14.68	1.134
2	Arjungenin	29.01	0.659
3	Arjunic acid	34.78	0.421

## Data Availability

All data generated or analyzed during this study are included in this article. Further enquiries can be directed to the corresponding author.
